# A Rare Case of Myocarditis Mimicking ST-Elevation Myocardial Infarction

**DOI:** 10.7759/cureus.11671

**Published:** 2020-11-24

**Authors:** Ashen Fernando, Nirmal Guragai, Rahul Vasudev, Raja Pullatt, Preet Randhawa

**Affiliations:** 1 Cardiology, St. George's University School of Medicine, True Blue, GRD; 2 Cardiology, Saint Joseph's University Medical Center, Paterson, USA; 3 Cardiology, Saint Michael's Medical Center, Newark, USA

**Keywords:** myocarditis, viral myocarditis, dilated cardiomyopathy, st-elevation myocardial infarction

## Abstract

Myocarditis is caused by acute injury and inflammation of cardiac myocytes and is most commonly caused by a viral infection. Myocarditis remains a rare diagnosis and manifests with a wide spectrum of non-specific symptoms that include chest pain, dyspnea, and palpitations associated with electrocardiographic abnormalities that resemble that of ST-elevation myocardial infarction (STEMI). Therefore, clinical diagnosis is often challenging and is often misdiagnosed. We present a case of a 22-year-old male who presented with left-sided non-radiating chest pain associated with shortness of breath, elevated troponin of 3.2 ng/ml (<0.03 ng/ml). Electrocardiogram (ECG) and cardiac echocardiogram revealed ST-segment elevations in the anterolateral leads and an ejection fraction of 35%, respectively. The patient was initially suspected of having a STEMI; however, cardiac catheterization revealed non-obstructed coronary arteries. Due to elevated inflammatory markers, the patient was then started on colchicine for suspected myocarditis and had complete resolution of symptoms one week after. This case highlights that a high index of clinical suspicion and prompt diagnosis is necessary to prevent any delays in appropriate therapy for myocarditis.

## Introduction

Myocarditis is an inflammatory disease of the myocardium that usually occurs after a viral infection. The disease can present with a wide spectrum of non-specific symptoms and sometimes mimics that of a myocardial infarction, which makes diagnosis challenging [1]. Myocyte injury and necrosis can result in repolarization changes on electrocardiogram (ECG) and sometimes are shown to have ST-segment elevations in the absence of intracoronary obstruction [2,3]. Here we present a case of myocarditis that presented with ST-segment elevation in the anterolateral and reciprocal ST-segment changes in the inferior leads in the absence of coronary artery obstruction or coronary spasm. The presenting history of the patient and the results of conventional diagnostic tests, such as ECG and biomarkers, are sometimes indistinguishable from acute ST-segment elevation myocardial infarction (STEMI), misdirecting the diagnosis and treatment decisions.

## Case presentation

**Figure 1:**
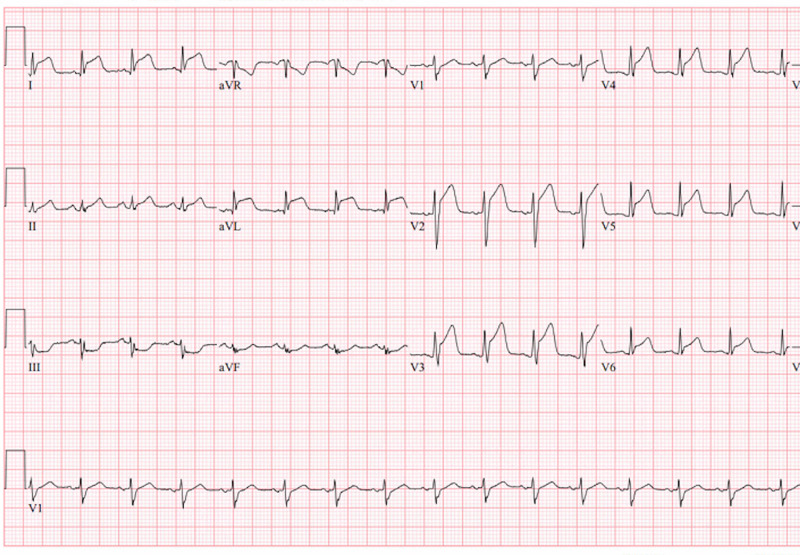
Electrocardiogram showing ST-segment elevation in leads V2-V5, I, aVL and reciprocal ST-segment depression in leads III and aVF

A 22-year-old male with no significant past medical history presented to the emergency room with a two-day history of left-sided chest pain that started after heavy exertion. He described the chest pain as dull and without any radiation. The pain was associated with shortness of breath. He denied cough, diaphoresis, or trauma. Laboratory tests on admission were significant for a troponin level of 3.2 ng/ml (<0.03 ng/ml). Initial ECG revealed ST-segment elevation in the anterolateral leads along with ST-segment depression in the inferior leads (Figure 1).

**Figure 2:**
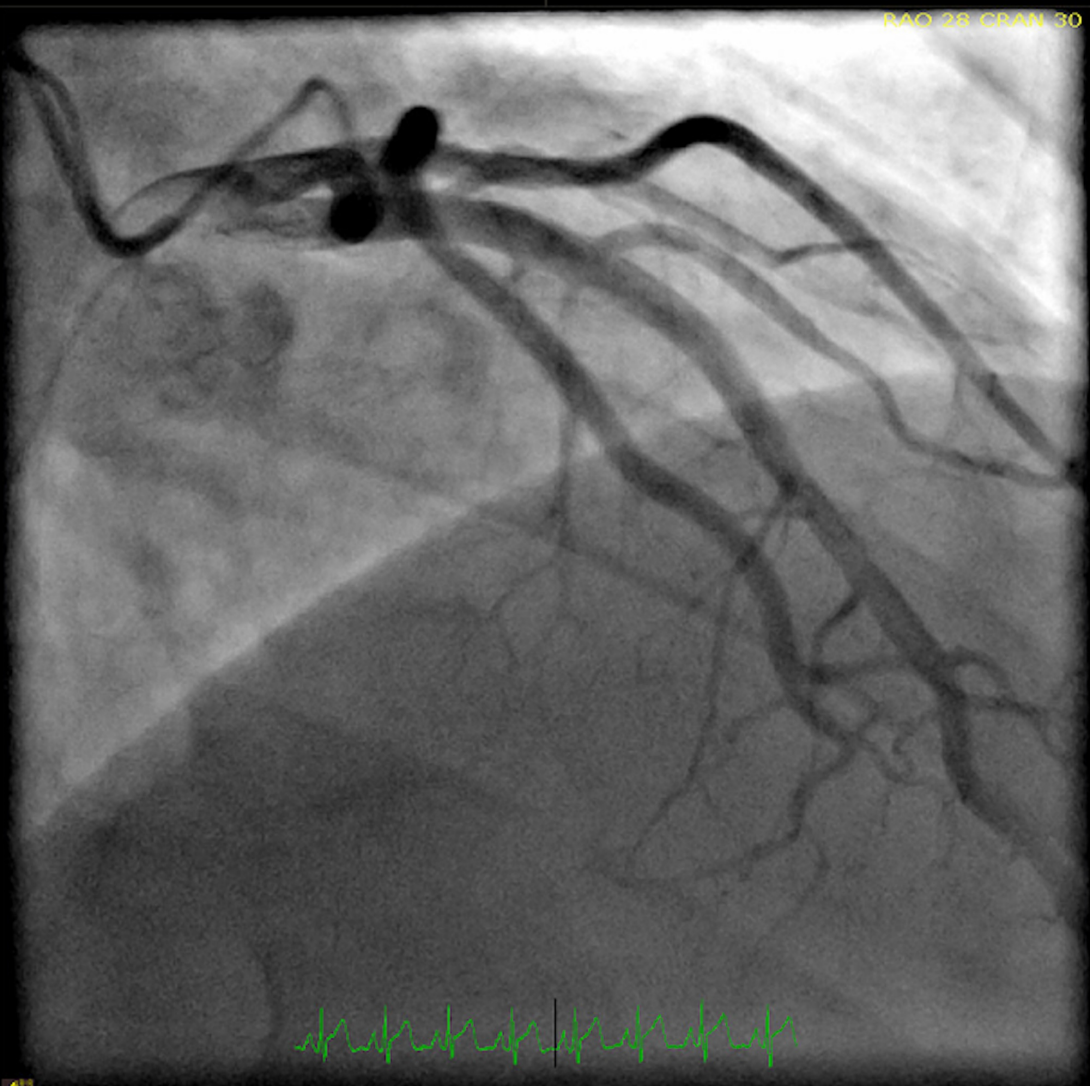
Left heart cardiac catheterization showing non-obstructed left coronary artery

**Figure 3:**
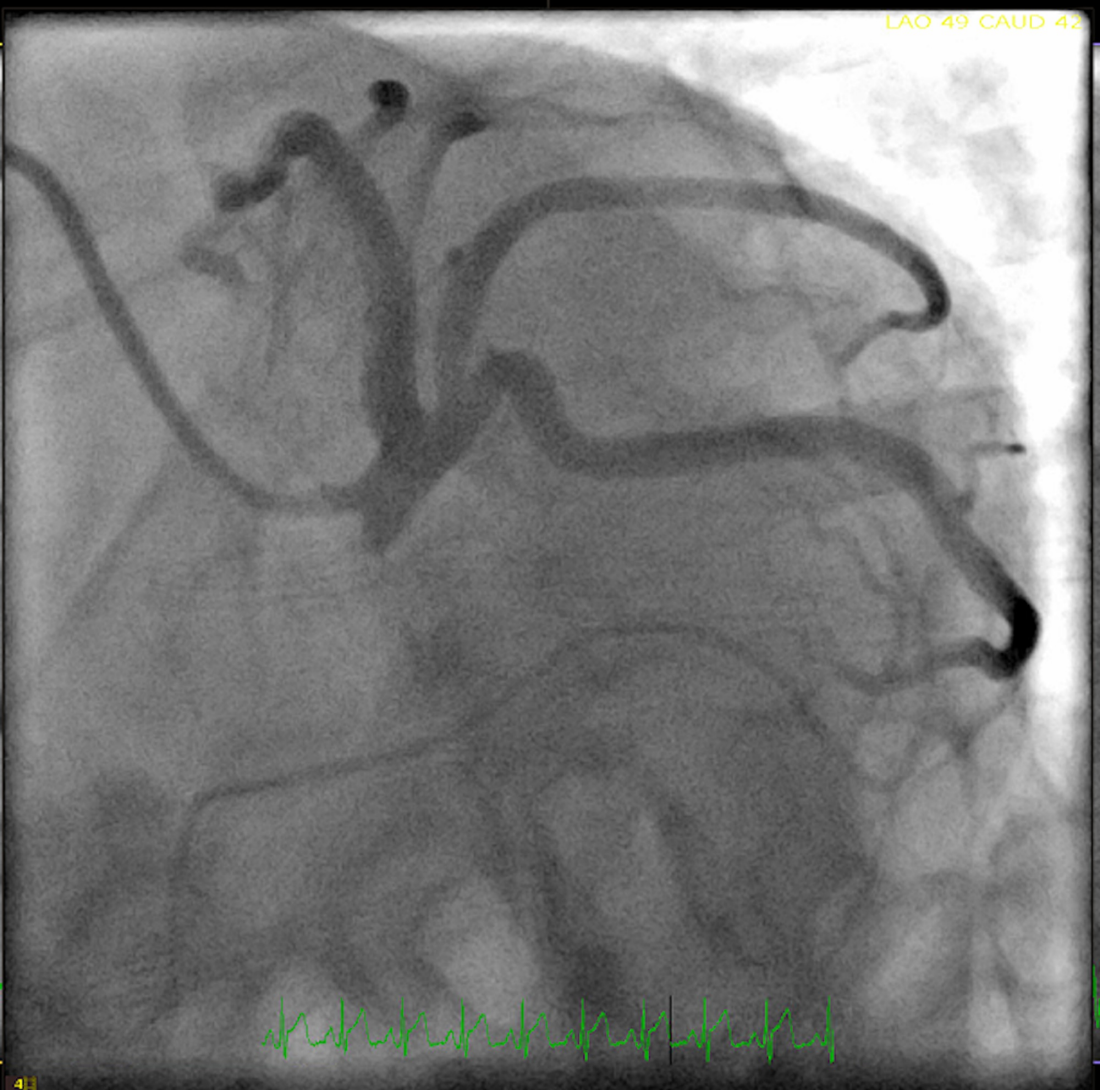
Left heart cardiac catheterization showing non-obstructed left coronary artery

**Figure 4:**
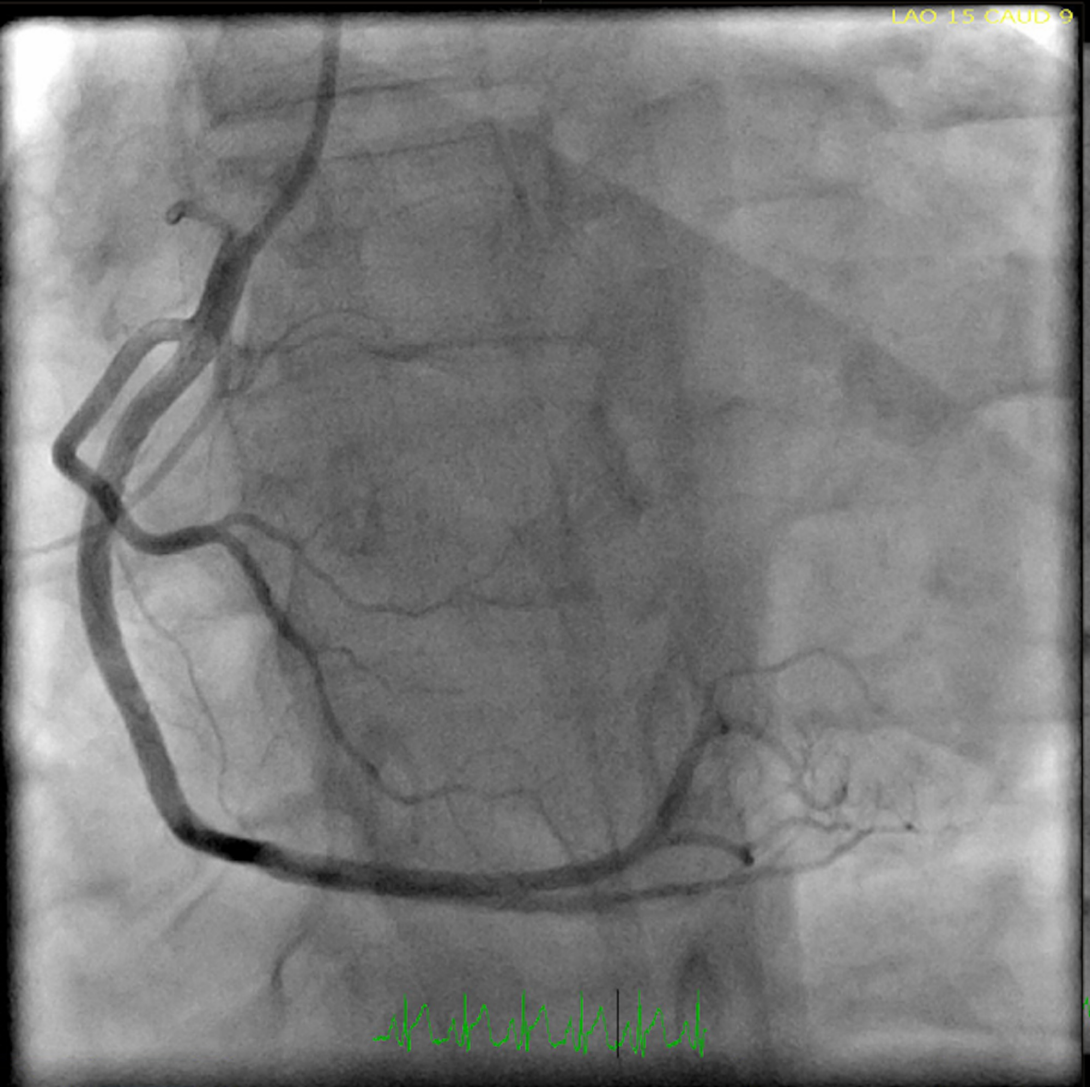
Left heart cardiac catheterization showing non-obstructed right coronary artery

Additionally, a 2D echocardiogram showed a left ventricular ejection fraction of 30-35% with global hypokinesia. The differential diagnosis at this time was broad and included acute coronary syndrome, coronary vasospasm, spontaneous coronary dissection, and myopericarditis. Given the classic ECG findings and elevated troponin, cardiac catheterization was performed. However, catheterization revealed non-obstructed coronary arteries (Figures 2-4).

Upon further investigation, the patient was noted to have elevated markers of inflammation, including erythrocyte sedimentation rate and C-reactive protein. The patient was scheduled for cardiac magnetic resonance imaging (CMRI); however, he did not consent. He was started on beta-blockers, angiotensin-converting enzyme (ACE) inhibitors, and colchicine for a suspected diagnosis of viral myocarditis. A follow-up one week later showed complete resolution of symptoms. The patient was discharged from the hospital but lost to follow up.

## Discussion

Myocarditis is the inflammation of the myocardium with necrosis and degeneration of adjacent myocytes [1]. Acute injury to the cardiac myocytes leads to the activation of the innate and humoral immune responses, which consequently leads to inflammation [2]. The incidence of myocarditis is estimated to be between 10 to 20 cases per 100,000 persons [1]. Furthermore, it has been reported that myocarditis often presents in younger patients with a paucity of conventional risk factors for accelerated atherosclerosis [2,3]. Myocarditis has an extremely low rate of diagnosis in clinical practice accounting for 1-9% of autopsies in general and 3-12% of sudden cardiac death in adults [4]. Viral infection is the most common cause of myocarditis, with adenovirus and enterovirus being the most frequently identified viruses [5,6]. Other etiologies include bacterial infections, protozoal infections, toxins, drug reactions, autoimmune-mediated (lupus, sarcoidosis, lymphocytic, and giant cell myocarditis), and malignancy [1,5-7].

The presentation of myocarditis can be highly variable, ranging from mild symptoms of chest pain, dyspnea, and palpitations associated with ECG abnormalities to life-threatening cardiogenic shock and ventricular arrhythmias [1,8,9]. A reported 20-30% of cases with diagnosed myocarditis can progress to dilated cardiomyopathy. Therefore, early diagnosis of myocarditis is crucial to improve patient outcomes, and this requires a high level of clinical suspicion along with appropriate investigations. Currently, the only available method for definitive diagnosis of myocarditis is an endomyocardial biopsy (EMB) [1]. Furthermore, immunohistochemistry characterization of the inflammatory infiltrate increases the diagnostic accuracy of EMB [9]. However, this is infrequently performed due to the invasive nature of the procedure and the probability of sampling errors caused by the characteristic patchy inflammation [2,10]. The more commonly performed studies include ECG, echocardiography, cardiac magnetic resonance imaging (CMRI) [11]. Markers of inflammation, viral serology, and cardiac biomarkers that include troponins and creatine kinase may also help confirm the diagnosis [1,9].

CMRI has emerged as one of the most useful tools in diagnosing myocarditis as it enables non-invasive visualization of myocardial inflammation. Additionally, it can identify areas of edema, scarring, fibrosis, and inflammation with a reported sensitivity and specificity of 100% and 90%, respectively [2,5,10,12]. However, the use of CMRI is limited in a considerable proportion of patients due to its high expense. Cardiac computed tomography (CT) is an alternative imaging tool with similar diagnostic accuracy and additional benefits, including better evaluation of myocardial inflammation, the feasibility of coronary artery anatomy investigation, and lower expense [10]. Although there are no specific echocardiographic findings of myocarditis, it can be used to exclude other causes of heart failure and intracavitary thrombi. 

The most common ECG findings are non-specific T wave changes [6,13]. Other less frequent changes include ST-segment elevation, ST-segment depression, PR segment depression, pathological Q-waves, and variable atrioventricular blocks [1,3]. Myocarditis mimicking ST-elevation myocardial infarction is rare, with an estimated clinical diagnostic incidence of 0.17 per 1000 man-years [4]. Nakashima et al. stated that myocarditis with pericardial involvement might contribute to ST elevations on ECG [13]. Furthermore, cell membrane leakage, accumulation of bioproducts, and a decrease in the inflamed myocardial tissue's oxygenation may further contribute to ST-segment changes [10,13]. The patient presented in this case underwent cardiac catheterization to rule out the possibility of an intracoronary thrombus or coronary artery spasm causing myocardial infarction, but cardiac catheterization revealed no obstruction or spasm. Therefore, ST-segment elevations in the absence of intracoronary thrombus or spasms and resolution of symptoms with the administration of colchicine make myocarditis the most likely diagnosis in this case. 

Regarding treatment, patients with mild symptoms of myocarditis improve spontaneously, and these patients can be managed with supplemental oxygen, optimization of fluid status, and reducing the burden on the heart while preserving left ventricular function [12]. This involves the restriction of physical activity and withholding of all cardiotoxic drugs. If the patient is symptomatic, treatment with diuretics, beta-blockers, ACE inhibitors, and angiotensin receptor blockers can be considered [8]. A variety of intravenous immunoglobulins, immunosuppressive agents, and interferon-beta have been associated with significant improvement of clinical outcomes, including viral clearance and improved left ventricular function [3,12]. Non-steroidal anti-inflammatory drugs, however, should be avoided as they can impede healing of the myocardium and exacerbate the inflammatory process [1]. On the other hand, patients with severe heart failure may benefit from mechanical circulatory support, such as an intra-aortic balloon pump or a left ventricular assist device [1-3,12]. If patients present with severe refractory ventricular arrhythmias or atrioventricular blocks, they may require antiarrhythmic medication or the insertion of implantable cardioverter defibrillators or temporary pacemakers [1,2]. 

Even though CMRI could not be performed in this patient, given the clinical presentation, unremarkable coronary angiogram, elevated markers of inflammation and cardiac biomarkers, myocarditis was the most likely diagnosis. Improvement of the patient's symptoms after starting an anti-inflammatory agent suggested an underlying inflammatory process and further supported the diagnosis of myocarditis.

## Conclusions

Myocarditis is not always a clear diagnosis, and this case highlights the diagnostic challenge of acute myocarditis. It can sometimes present with chest pain and localized ST-segment elevation ECG changes that mimic myocardial infarction. If a young patient with no significant cardiovascular risk factors presents to the emergency department with chest pain and ST-segment elevation on ECG, myocarditis should be an important differential diagnosis. A thorough history, physical examination, and appropriate investigations are crucial for the timely diagnosis and management of myocarditis.
